# Inhibition of IL-17 signaling in macrophages underlies the anti-arthritic effects of halofuginone hydrobromide: Network pharmacology, molecular docking, and experimental validation

**DOI:** 10.1186/s12906-024-04397-2

**Published:** 2024-02-27

**Authors:** Junping Zhu, Jiaming Wei, Ye Lin, Yuanyuan Tang, Zhaoli Su, Liqing Li, Bin Liu, Xiong Cai

**Affiliations:** 1 Department of Rheumatology, First Hospital, School of Chinese Medical Sciences, Hunan University of Chinese Medicine, Changsha, Hunan 410208 China; 2 College of Biology, Hunan University, Changsha, Hunan 410082 China; 3 The Central Research Laboratory, Hunan Traditional Chinese Medical College, Zhuzhou, China; 4 Guangxi Provincial Key Laboratory of Preventive and Therapeutic Research in Prevalent Diseases in West Guangxi, Youjiang Medical University for Nationalities, Baise, Guangxi 533000 China

**Keywords:** Halofuginone hydrobromide, Rheumatoid arthritis, Network pharmacology, Molecular docking, Experimental validation

## Abstract

**Background:**

Rheumatoid arthritis (RA) is a prevalent autoimmune disease marked by chronic synovitis as well as cartilage and bone destruction. Halofuginone hydrobromide (HF), a bioactive compound derived from the Chinese herbal plant *Dichroa febrifuga* Lour., has demonstrated substantial anti-arthritic effects in RA. Nevertheless, the molecular mechanisms responsible for the anti-RA effects of HF remain unclear.

**Methods:**

This study employed a combination of network pharmacology, molecular docking, and experimental validation to investigate potential targets of HF in RA.

**Results:**

Network pharmacology analyses identified 109 differentially expressed genes (DEGs) resulting from HF treatment in RA. Gene Ontology (GO) and Kyoto Encyclopedia of Genes and Genomes (KEGG) analyses unveiled a robust association between these DEGs and the IL-17 signaling pathway. Subsequently, a protein-protein interaction (PPI) network analysis revealed 10 core DEGs, that is, EGFR, MMP9, TLR4, ESR1, MMP2, PPARG, MAPK1, JAK2, STAT1, and MAPK8. Among them, MMP9 displayed the greatest binding energy for HF. In an in vitro assay, HF significantly inhibited the activity of inflammatory macrophages, and regulated the IL-17 signaling pathway by decreasing the levels of IL-17 C, p-NF-κB, and MMP9.

**Conclusion:**

In summary, these findings suggest that HF has the potential to inhibit the activation of inflammatory macrophages through its regulation of the IL-17 signaling pathway, underscoring its potential in the suppression of immune-mediated inflammation in RA.

**Supplementary Information:**

The online version contains supplementary material available at 10.1186/s12906-024-04397-2.

## Introduction

Rheumatoid arthritis (RA) is an autoimmune disorder characterized by chronic synovitis, progressive cartilage and bone erosion, synovial hyperplasia, joint deformity, and disability [1]. The global prevalence of RA is estimated to range between 0.25% and 1% [2]. Immunoinflammatory cell infiltration and serological markers form the RA immune-inflammatory environment [3]. In the immune-inflammatory environment, the activation of macrophages plays a crucial role in triggering RA by promoting the excessive production of pro-inflammatory mediators and chemokines. These factors activate various immune and non-immune cells, leading to chronic inflammation, tissue damage, and pain [4]. If left untreated, this destructive inflammation can stimulate abnormal synovium proliferation and irreversible bone destruction. Various therapeutic agents have primarily been used to treat RA, including disease-modifying antirheumatic drugs, glucocorticoids, nonsteroidal anti-inflammatory drugs, and inflammatory cytokine inhibitors [5]. However, these medications neither cure nor prevent RA, and their association with adverse effects often result in treatment discontinuation. Approximately half of patients discontinue treatment due to poor efficacy or adverse reactions, and 20–30% of are unresponsive to all treatment drugs [6]. Consequently, there is an urgent need to explore alternative drugs for the treatment and management of RA.

Over the past few decades, natural products have gained significant prominence in pharmaceutical research and development, particularly in the context of autoimmune diseases. Halofuginone hydrobromide (HF) is a natural plant alkaloid derived from the roots of *Dichroa febrifuga* Lour [7]. Zhan et al. (2017) reported its anti-inflammatory, immune regulatory, anti-fibrotic, and antibacterial properties [8]. In mice with collagen-induced arthritis (CIA), HF demonstrated the potential to improve autoimmune arthritis by regulating the balance between Th17 and Treg cells and inhibiting osteoclastogenesis [9]. Additionally, HF hinder the TNF-α-induced migration and proliferation of RA fibroblast-like synoviocytes (RAFLS) [10]. These findings suggest that HF may ameliorate RA by mediating immune and inflammatory responses, although its precise mechanisms of action are yet to be elucidated. A recent study (2024) demonstrated that HF exerted anti-inflammatory effect through activating GCN2 to regulate macrophage polarization, reducing the proportion of M1 macrophages and the expression levels of pro-inflammatory genes Cx3cr1 and IL-6, and promoting the polarization of M2 macrophages and the expression of anti-inflammatory factors, including CD206, IL-10, and TGF-β [11]. Nevertheless, additional comprehensive research is required to elucidate the therapeutic mechanisms of HF in RA.

**Fig. 1:**
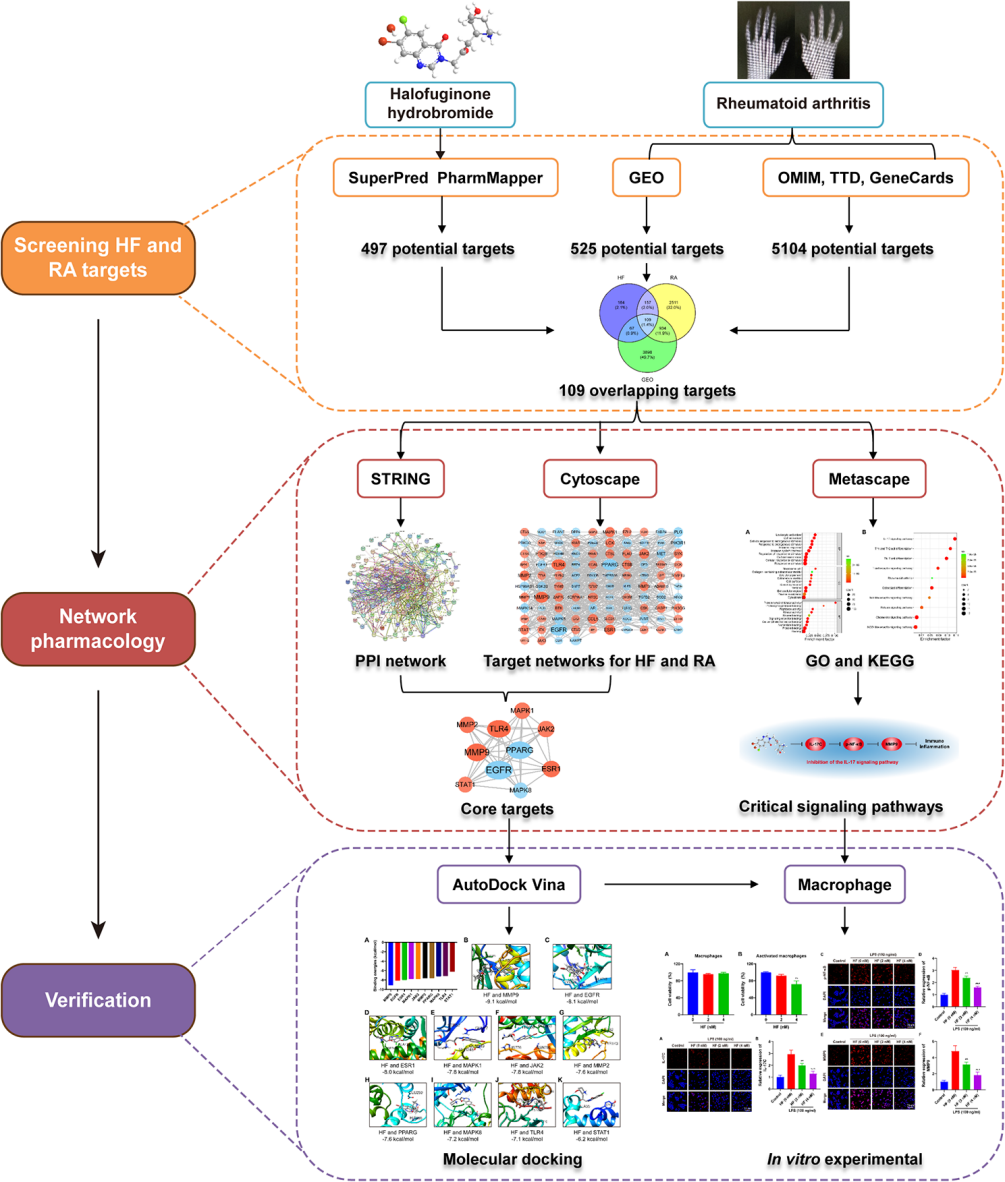
Technical strategy of the study

Network pharmacology is an innovative strategy for exploring novel drugs based on the therapeutic targets of herbs and compounds. It seeks to enhance clinical efficacy while comprehending the side effects and toxicity of these compounds. In recent practice of drug therapy mechanisms, network pharmacology is used to prioritize signaling pathways and key targets by constructing databases and conducting network and enrichment analyses. Furthermore, the reliability of predicted results is ensured through molecular docking and experimental validation [12]. Network pharmacology marks a shift from the traditional research paradigm of “one drug, one disease, one gene” toward a novel “network pharmacology” framework [13]. This study had three principal aims: (1) to identify potential targets of HF and differentially expressed genes (DEGs) in RA synovial tissue, (2) to analyze the underlying mechanisms of HF in RA using network pharmacology and molecular docking, and (3) to validate the anti-inflammatory effects and associated pathways of HF. These findings could contribute to the development of new drugs for the treatment of RA and clarify the molecular mechanisms of HF in the treatment of this disease. Figure 1 illustrates the technical strategies employed in this study.

## Materials and methods

### Chemical reagents

HF was procured from TopScience (Shanghai, China), subsequently dissolved in dimethyl sulfoxide (DMSO), and then further diluted in Dulbecco’s modified Eagle medium (DMEM). Fetal bovine serum (FBS), DMEM, trypsin, and antibiotics (streptomycin/penicillin) were purchased from Gibco. 3-[4,5-dimethyl-thiazol-2-yl]-2,5-diphenyl-tetrazolium bromide (MTT) and DMSO were obtained from Beijing Suolaibao Technology Co., Ltd. (Beijing, China). Interleukin-17 C (IL-17 C) and phosphorylated nuclear factor-κB (p-NF-κB) were procured from Cell Signaling, while matrix metalloproteinase-9 (MMP-9) was sourced from Proteintech Group Inc. (Wuhan, China). Alexa Fluor 647-labeled Goat Anti-Rabbit IgG (H + L) was acquired from Beyotime (Jiangsu, China).

### HF target prediction and RA-related target screening

#### Target prediction of HF

The chemical structure and simplified molecular input line entry specification (SMILES) of HF were retrieved from PubChem (https://pubchem.ncbi.nlm.nih.gov/compound/442428) [14]. HF target prediction was conducted using the PharmMapper (http://www.lilab-ecust.cn/pharmmapper/) [15] and SuperPred (https://prediction.charite.de/subpages/ target_prediction.php) [16] databases, with the species restricted to “Homo sapiens”.

#### Acquisition of RA-related targets

RA-related targets were obtained from Online Mendelian Inheritance in Man (OMIM) (https://www.omim.org/) [17], GeneCards (https://www.genecards.org/) [18], and the Therapeutic Target Database (http://db.idrblab.net/ttd/) [19]. A keyword search for “Rheumatoid arthritis” was conducted to identify relevant genes. The collected targets were consolidated, and duplicate entries were eliminated to compile disease targets.

#### Acquisition of RA-related gene expression data

Gene expression data (GSE55235, and GSE77298) from synovial tissues of patients with RA and healthy donors were obtained from the Gene Expression Omnibus (GEO) dataset of the National Center for Biotechnology Information (https://www.ncbi.nlm.nih.gov/geo/) [20]. The GSE55235 dataset comprised gene expression data from 10 synovial tissue samples from healthy donors and 10 samples from patients with RA. The GSE77298 dataset constituted gene expression data from seven synovial tissue samples from healthy donors and 17 samples from patients with RA. GEO2R was employed to identify DEGs with *P* < 0.05 and |logFC| ≥ 1, to obtain DEGs between normal tissues and RA. Volcano maps of the DEGs were subsequently generated.

#### Cross-tabulation of drugs, diseases, and GEO

Using the Venny online tool (https://bioinfogp.cnb.csic.es/tools/venny/index.html), the intersections of drug targets, disease targets, and GEO genes were obtained.

### GO and KEGG pathway enrichment analyses

To attain a more profound understanding of the function and significance of the shared genes in the signaling pathways, we uploaded the intersecting target genes from HF and RA to an annotation, visualization, and integrated discovery database, namely Metascape (https://metascape.org/gp/ index.html#/main/step1) [21]. This facilitated the determination of functional term enrichment through Gene Ontology (GO) enrichment analysis, encompassing Biological Process (BP), Molecular Function (MF), and Cellular Component (CC) analyses. Furthermore, we harnessed the KEGG knowledge base [22], a bioinformatics resource, to discern significantly altered metabolic pathways enriched in the gene list. Subsequently, we visualized the results of the GO enrichment analysis (*p* < 0.05) and KEGG pathway analysis (*p* < 0.05) through a bioinformatics platform (http://www.bioinformatics.com.cn/).

### Protein-protein interaction network analysis

The protein-protein interaction (PPI) network of the target genes was extracted from the Search Tool for the Retrieval of Interacting Genes/Proteins (STRING [23]; https://string-db.org/). This PPI network was then rendered using Cytoscape version 3.9.0 (http://www.cytoscape.org/). Within the PPI network, the nodes represented the target proteins, and the edges signified the predicted or validated interactions among these proteins. We conducted topological analysis of the target genes utilizing the CytoNCA plug-in [24] of Cytoscape. The target proteins were independently filtered based on several centrality metrics, including Betweenness Centrality (BC), Closeness Centrality (CCT), Degree Centrality (DC), Subgraph Centrality (SC), Eigenvector Centrality (EC), Information Centrality (IC), Local Average Connectivity-Based Method (LAC), and Network Centrality (NC), which were calculated using the CytoNCA plug-in.

### Molecular docking

We obtained the structure of HF, a small molecule ligand, from PubChem (https://pubchem.ncbi.nlm.nih.gov/#query=Halofuginone%20hydrobromide). The crystal structures of the key targets (macromolecular receptors) were retrieved from the RCSB Protein Data Bank (https://www.rcsb.org/) [25]. The protein and HF structures were imported into the AutoDock Vina plug-in within the Chimera software for processing and conducting molecular docking. This included the calculation of intermolecular docking affinity and subsequent visualization. Finally, we selected the target component with the lowest affinity score for further exploration.

### Cell viability assay

An MTT assay was used to detect HF toxicity in macrophages. RAW264.7 cells or RAW264.7 cells activated with lipopolysaccharides (LPS, 100 ng/mL) (8 × 10^3^/well) were seeded in 96-well plates and cultured for 24 h, followed by the addition of fresh media containing PBS and HF at concentrations of 2 nM or 4 nM for 24 h. Then, 10 µL of 5 mg/mL MTT solution with 90 µL of DMEM was added to each well and incubated for 4 h. Lastly, the media was discarded, and 100 µL of DMSO was added to each well. The optical density (OD) was measured at a wavelength of 490 nm using a microplate reader. Each experiment was repeated thrice. Cell viability was calculated using the following equation:

1 $${{O{D_{experimental\,group}}\, - \,O{D_{blank}}} \over {O{D_{control\,group}}\, - \,O{D_{blank}}}}$$

### Immunofluorescence analysis

To perform cellular immunofluorescence, we first fixed and permeabilized the treated cells. Following this, they were subjected to an overnight incubation with the primary antibody. Subsequently, the cells were exposed/incubated to a fluorescent secondary antibody, Alexa Fluor 647-labeled Goat Anti-Rabbit IgG (H + L), at a dilution of 1:200, for 1 h at 37℃ in the absence of light. Finally, DAPI was introduced to stain the nuclei, and the cells were visualized using an FV1200 Confocal Laser Scanning Microscope (Olympus, Tokyo, Japan).

### Ethics approval

This project was approved by the Medical Ethics Committee of Hunan University of Chinese Medicine.

### Statistical analysis

The data are expressed as the mean ± standard deviation. All statistical analyses were performed using GraphPad Prism software version 8 (GraphPad Software, La Jolla, CA, United States). The differences among multiple groups were analyzed using analysis of variance (ANOVA), where a *P* < 0.05 was considered statistically significant.

## Results

### Target screening of HF and RA

**Fig. 2:**
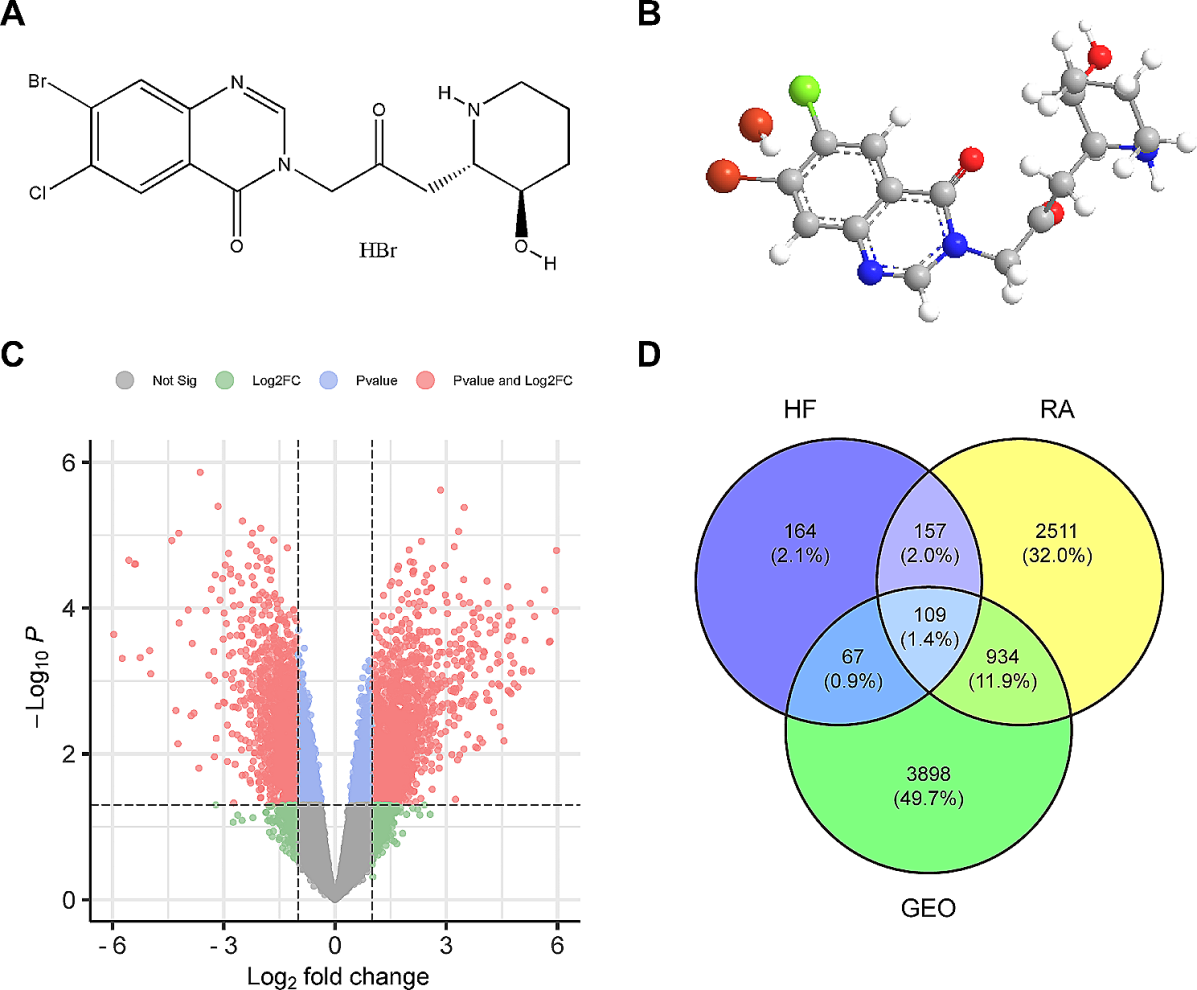
Information on the potential targets of HF and RA. **(A)** The 2D chemical structure of HF. **(B)** The 3D chemical structure of HF. **(C)** GEO Volcano Map. Red is significant for both FC and P, Blue is significant for P, Green is significant for FC, Grey is not significant. Gene expression in normal subjects vs. patients with RA. **(D)** Venn diagram of HF, RA, and GEO.

The 2D and 3D structures of HF were acquired from PubChem (Fig. 2A and B). The potential targets of HF were identified using the PharmMapper and SuperPred databases, resulting in a total of 497 targets after rigorous screening, deduplication, and integration. Disease-related targets were further identified through thorough screening using the GeneCards, OMIM, and TTD databases, yielding a set of 3,711 targets. Additionally, we extracted data on 5,104 distinct genes from the GEO database, highlighting gene expression differences between normal individuals and those with RA. Among these, 2,028 genes were found to be upregulated, while 3,076 were downregulated. The volcano plot (Fig. 2C) provides a clear visualization of the marked genetic distinctions present in synovial tissues between patients with RA and individuals without the condition. Through a comprehensive integration of drug, disease, and GEO targets, we successfully identified 109 overlapping targets, as illustrated in Fig. 2D, using a Venn diagram.

### GO and KEGG analysis

**Fig. 3:**
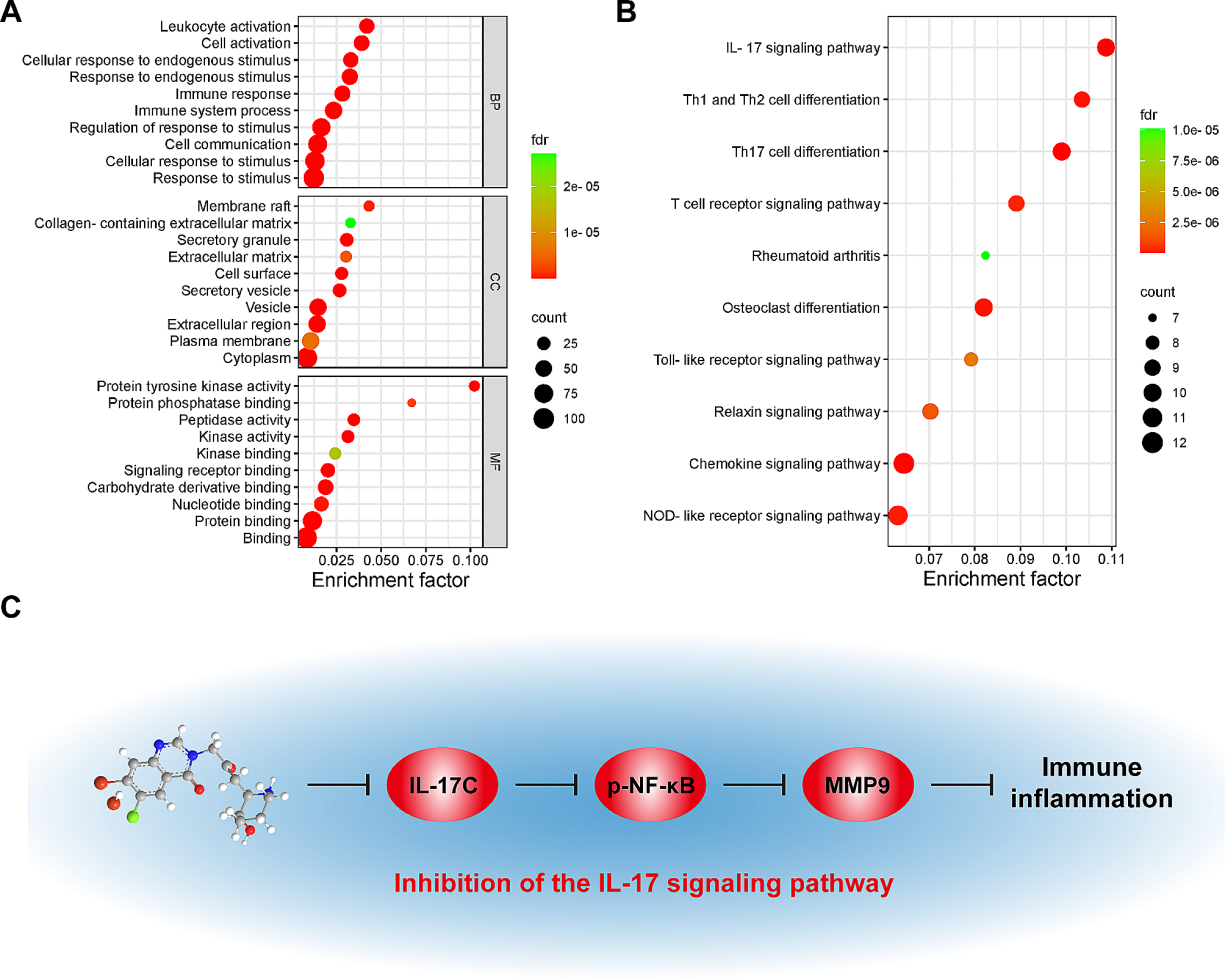
GO and KEGG analysis of the target proteins of HF against RA. **(A)** GO, BP, CC, and MF enrichment analysis of the intersection targets. The intensity of the color represents the fdr value, and the bubble size corresponds to the number of genes. **(B)** KEGG enrichment analysis of the intersection targets. The intensity of the color represents the fdr value. **(C)** IL-17 signaling pathway map

The 109 targets of common significance for drugs and diseases were analyzed using GO and KEGG analyses. Figure 3A and Tables S1–S3 show the most important GO categories in RA. The targets of 1017 BPs were mainly enriched in response to oxygen-containing compounds, organic substances, chemicals, cellular response to chemical stimuli, and nitrogen compounds. The targets of the 59 enriched CCs were mainly developed in the extracellular regions, extracellular spaces, vesicles, ficolin-1-rich granule lumens, and secretory granules. The targets of 87 enriched MFs were mainly enriched in catalytic activity, identical protein binding, catalytic activity acting on a protein, small-molecule binding, and protein binding. Enrichment analysis of the core targets provided guidance for future experimental validation.

KEGG analysis of the common targets in HF and RA identified 133 pathways. By creating a pathway-target network, the top 30 KEGG pathways were predominantly enriched for the IL-17 signaling pathway (hsa04657), Th17 cell differentiation (hsa04659), osteoclast differentiation (hsa04380), Th1 and Th2 cell differentiation (hsa04658), and the T cell receptor (TCR) signaling pathway (hsa04660) (Fig. 3B, Table S4). Notably, among these pathways, the top-ranked in relation to RA was the IL-17 signaling pathway, which has been reported to aggravate RA when activated [26]. This process involves the expression of IL-17, NF-κB, and MMP [27, 28]. Regulation of these targets can alleviate RA. Previous studies have shown that HF can inhibit MMP9 activity in the proliferation of TNF-α-induced RAFLS [10]. HF alleviates autoimmune diseases by inhibiting the expression of IL-17 [29]. Moreover, HF had an inhibitory effect on NF-κB in alleviating intervertebral disc degeneration [30]. We thus propose that the potential mechanism of HF in relieving RA may be related to IL-17, NF-κB, and MMP9 in the IL-17 signaling pathway (Fig. 3C).

### PPI network construction and screening of key targets

**Fig. 4:**
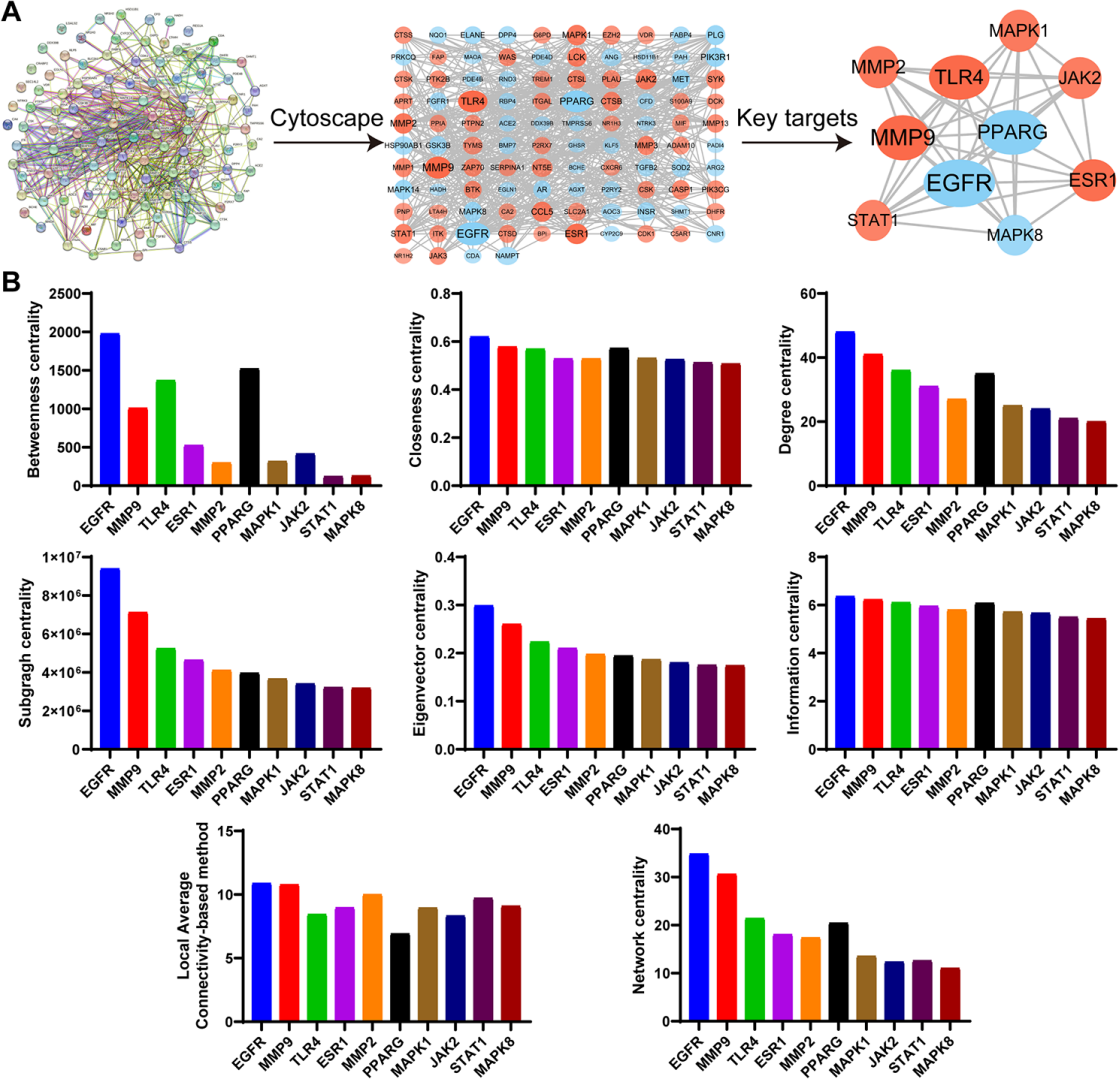
Protein–protein interaction (PPI) network construction of the target proteins of HF against RA. **(A)** PPI network and key core targets screening. The more connections, the stronger the correlation with the therapeutic target. The larger the DC, the larger the label font size and the darker the color. The larger the BC, the wider the circle. **(B)** Detailed information on the topological networks of the top 10 targets

The HF and RA intersection targets were imported into the STRING database. The obtained protein interaction network was imported into Cytoscape for topological analysis (Fig. 4A). The analysis revealed that the network consisted of 104 nodes and 581 edges. Using the CytoNCA plugin to identify the key core targets (Fig. 4A), we found that EGFR, MMP9, TLR4, ESR1, MMP2, PPARG, MAPK1, JAK2, STAT1, and MAPK8 were the top 10 targets for RA treatment. Detailed information on the targets ranked according to the key target values of BC, CCT, DC, SC, EC, IC, LAC, and NC is shown in Fig. 4B and Table S5.

### Molecular docking

**Fig. 5:**
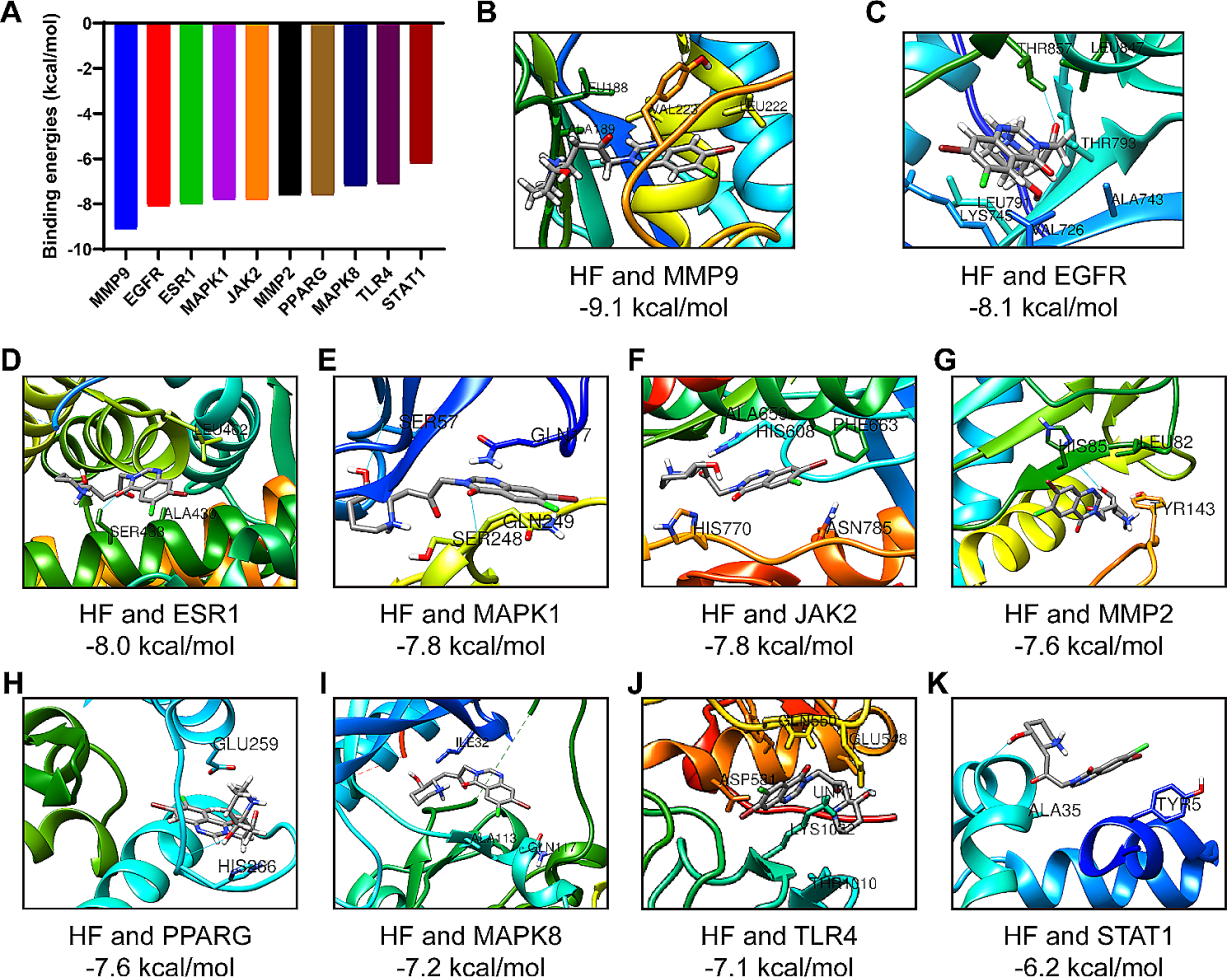
Binding energies of HF and core targets (kcal/mol). **(A)** The binding energy results of molecular docking. **(B)** Docking pattern of HF with MMP9. **(C)** Docking pattern of HF with EGFR. **(D)** Docking pattern of HF with ESR1. **(E)** Docking pattern of HF with MAPK1. **(F)** Docking pattern of HF with JAK2. **(G)** Docking pattern of HF with MMP2. **(H)** Docking pattern of HF with PPARG. **(I)** Docking pattern of HF with MAPK8. **(J)** Docking pattern of HF with TLR4. **(K)** Docking pattern of HF with STAT1.

In this study, molecular docking of key targets to HF was performed using AutoDock Vina software, which evaluated the binding energy, a crucial indicator for verifying the stability of the protein and active ingredient conformation [31]. The results revealed significant negative docking energy values for HF with MMP9, EGFR, ESR1, MAPK1, JAK2, MMP2, PPARG, MAPK8, TLR4, and STAT1 (Fig. 5). The binding energies were then calculated to determine the level of compatibility between the small molecules and proteins, as lower binding energies indicate higher stability. The binding energies between HF and MMP9, EGFR, ESR1, MAPK1, JAK2, MMP2, PPARG, MAPK8, TLR4, or STAT1, were − 9.1, -8.1, -8.0, -7.8, -7.8, -7.6, -7.6, -7.2, -7.1, or -6.2 kcal/mol, respectively (Fig. 5). The detailed information of core targets is shown in Table S6. Moreover, as a crucial target of the IL-17 signaling pathway, MMP9, exhibited the highest binding affinity. These findings align with the results of the KEGG analysis, which further supports the significant role of the IL-17 signaling pathway in RA treatment using HF.

### HF decreases cell viability of activated macrophages

**Fig. 6:**
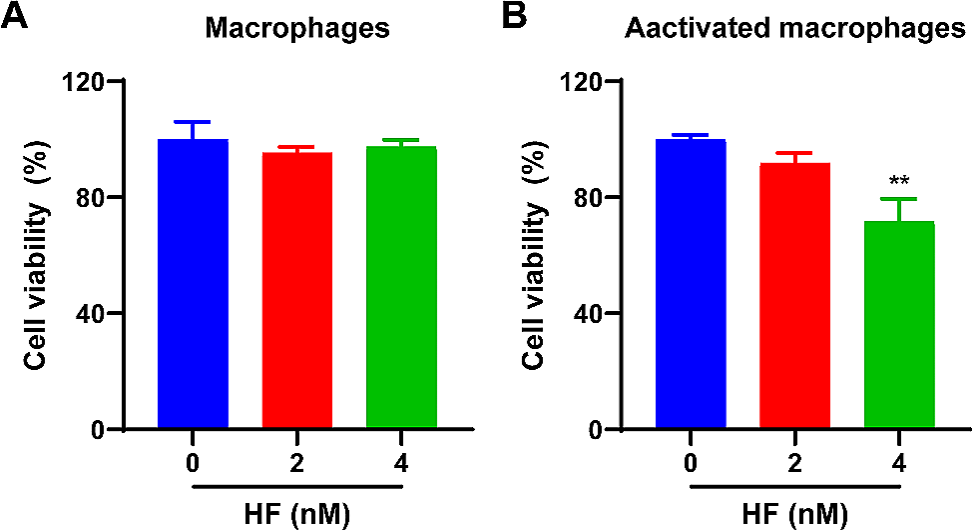
Cell viability of macrophages and activated macrophages were validated in vitro. **(A)** Cytotoxicity assay of macrophages with different concentrations of HF (0, 2, or 4 nM) for 24 h (*n* = 3 per group). **(B)** Effect of different concentrations of HF (0, 2, or 4 nM) on the viability of activated macrophages after co-incubation for 24 h (*n* = 3 per group). The data are represented as the means ± SD; * *p* < 0.05, ** *p* < 0.01

The cytotoxicity of HF was evaluated using macrophages (RAW 264.7). Macrophages are immune cells in the human body that engulf and degrade debris, dead cells, and foreign objects. They also play a crucial role in the inflammatory process in RA by secreting anti-inflammatory cytokines and maintaining normal joint function [32]. Figure 6A shows the ultralow toxicity of HF in macrophages, with over 90% cell viability.

A large number of macrophages are recruited and activated at the lesions sites. Activated macrophages secrete various proinflammatory cytokines that participate in immune inflammation, promoting synovial hyperplasia and joint destruction, thereby exacerbating RA. It is also serve as manifestations of immune-inflammatory functions, which can be used for in vitro study of the mechanism of immune inflammation in RA. To investigate the impact of HF on activated macrophages, a cell viability assay was conducted using MTT after exposure of the cells to 0, 2, or 4 nM HF for 24 h. Figure 6B shows that the cell viability of activated macrophages (treated with LPS at a concentration of 100 ng/mL for 24 h) decreased in a concentration-dependent manner.

### HF-RA pathway screening and key target validation

**Fig. 7:**
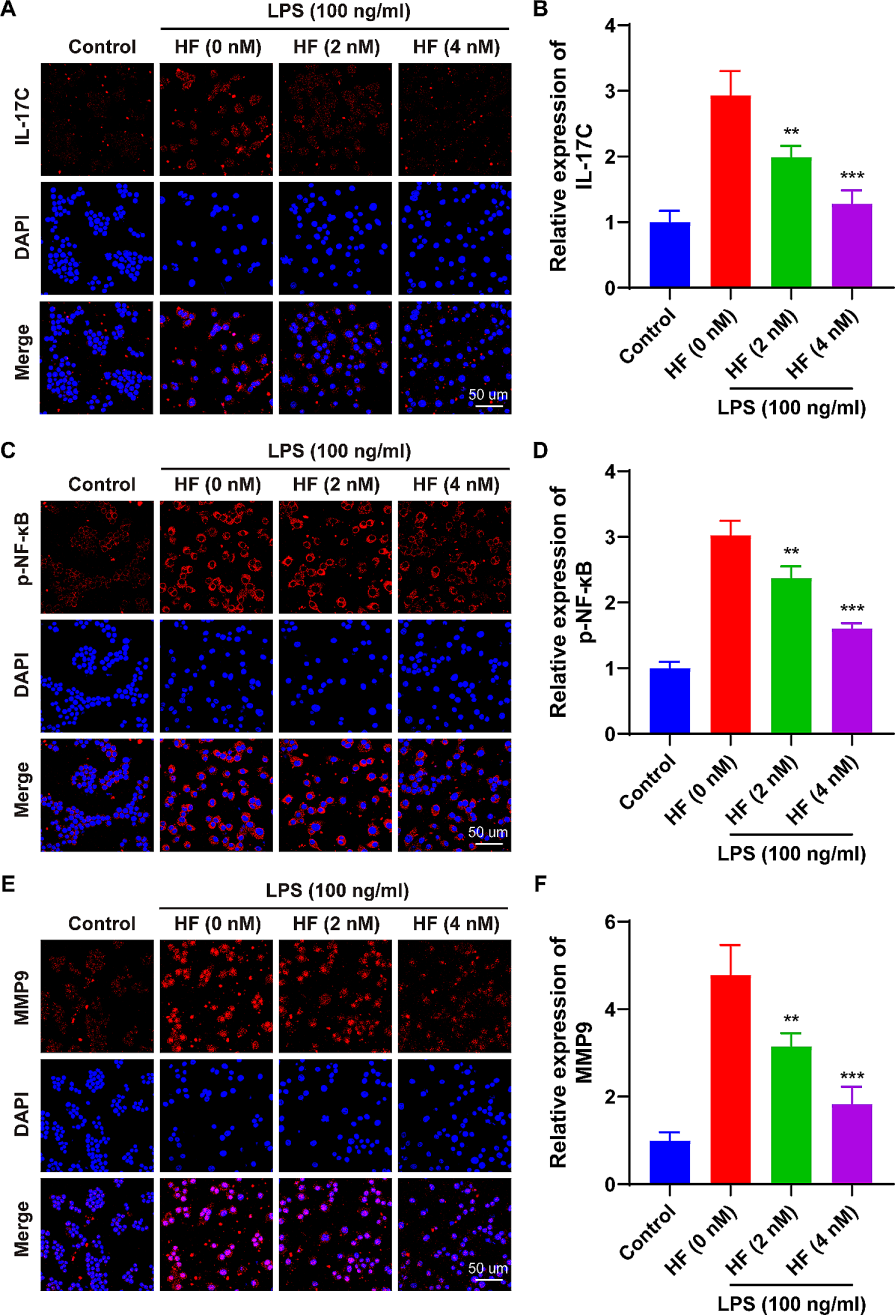
HF reduces the expression of IL-17 C, p-NF-κB, and MMP9 in the IL-17 signaling pathway of activated macrophages. **(A, B)** IL-17 C immunofluorescence expression in macrophages, along with the corresponding fluorescence intensity data (*n* = 3 per group). **(C, D)** p-NF-κB immunofluorescence expression in macrophages, along with the fluorescence intensity data (*n* = 3 per group). **(E, F)** MMP9 immunofluorescence expression in macrophages, along with the fluorescence intensity data (*n* = 3 per group). The data are represented as means ± SD; * *p* < 0.05, ** *p* < 0.01, *** *p* < 0.001

Based on the results of network pharmacology, we investigated the levels of IL-17 C, p-NF-κB, and MMP9 in normal macrophages (control) and activated macrophages (LPS, 100 ng/mL) co-incubated with 0, 2, or 4 nM HF for 24 h. As expected, LPS-induced macrophages exhibited increased levels of IL-17 C, p-NF-κB, and MMP9 after activation compared to normal macrophages. Notably, these levels were significantly reduced after co-incubation with HF compared to the LPS stimulation group. Both the 2 nM HF and 4 nM HF treatments reduced the expression of IL-17 C by ~ 32.1% and ~ 56.3% (Fig. 7A and B), the levels of p-NF-κB by ~ 21.5% and ~ 46.9% (Fig. 7C and D), and the expression of MMP9 by ~ 34.1% and ~ 61.6% (Fig. 7E and F), respectively. These results indicate that HF significantly inhibits the levels of IL-17 C, p-NF-κB, and MMP9 in the IL-17 signaling pathway of activated macrophages.

## Discussion

RA is a complex autoimmune inflammatory disease that affects the joints. The development of RA involves the activation of immune cells, particularly inflammatory macrophages, which play a significant role disease progression [2]. One characteristic of active RA is the presence of an elevated number of inflammatory macrophages. These in turn release proinflammatory cytokines, including IL-17, along with a substantial quantity of matrix metalloproteinases (MMPs). Current drug treatments for RA have limited efficacy and severe side effects. Therefore, it is crucial to explore new potential drugs and their mechanisms of action for the treatment and management of the disease. HF exerts anti-inflammatory and immunoregulatory properties by modulating T cell balance, inhibiting expression of MMPs in RAFLS, and attenuating inflammation in macrophages [9–11]. It has also been shown to exert beneficial effects in a mouse model of autoimmune arthritis. However, the underlying mechanism by which HF alleviates RA remains unclear. Network pharmacological analysis is a systematic and holistic approach that explores the therapeutic effects and underlying mechanisms of drugs by analyzing the network of drug-target interactions [12]. In this study, we utilized network pharmacology to investigate the therapeutic targets involved in RA treatment by HF. We conducted GO, KEGG pathway, and PPI network analyses to identify potential therapeutic targets and pathways. Experimental validation was performed to confirm the therapeutic effects of HF in RA.

First, we comprehensively integrated drugs, diseases, and GEO targets and identified a total of 109 therapeutic targets for HF and RA. Among them, EGFR, MMP9, TLR4, ESR1, MMP2, PPARG, MAPK1, JAK2, STAT1, and MAPK8 were the top 10 core targets in treating RA. Target-related enrichment analyses of GO and KEGG pathways revealed that the therapeutic efficacy of HF in RA involves multiple biological processes and pathways, with the top-ranked pathway being the IL-17 signaling pathway. Subsequently, molecular docking analysis indicated that HF had the highest binding affinity for MMP9. MMP9 is a key node in the IL-17 signaling pathway. This is consistent with the results of KEGG analysis and further supports the important role of the IL-17 signaling pathway in the treatment of RA using HF. In this study, in vitro experiments were conducted to verify KEGG pathway analysis results. The experimental results showed that HF significantly inhibited the viability of inflammatory macrophages, and regulated the IL-17 signaling pathway by downregulating the levels of IL-17 C, p-NF-κB, and MMP9.

In the IL-17 signaling pathway, activation of IL-17 C stimulates the production of NF-κB [28], subsequently inducing the production of MMPs, such as MMP9 [27]. Studies have shown that IL-17 promotes synovial inflammation, cartilage destruction, bone erosion, and synovial pannus formation by aggravating inflammation [33]. First, activation of IL-17 is a key factor in promoting inflammation, as it facilitates the immune response and enhances the activation of immune cells, particularly T cells and macrophages. These activated immune cells produce various inflammatory mediators such as chemokines, cytokines, growth factors, MMPs, and prostaglandins, which further attract and activate other inflammatory cells to form a positive feedback loop, exacerbating joint inflammation and bone destruction [33]. In addition, IL-17 plays a positive role in the formation of pannus, causing abnormal activity of the hematopoietic system, promoting the accumulation of immune cells in joint tissues, thereby triggering and worsening synovitis [34]. IL-17 C, a member of the IL-17 family, is highly expressed in synovial fluid mononuclear cells and PBMCs of patients with RA [35]. Previous studies have demonstrated that IL-17 C induces elevated levels of TNF-α and aggravates arthritis in CIA mice, and is positively correlated with the degree of inflammation and disease activity [35]. Therefore, NF-κB is activated in RA and stimulates the production of pro-inflammatory factors to promote inflammation. NF-κB also regulates the generation of MMPs. The produced MMPs, including MMP-9, directly exacerbate cartilage destruction and bone erosion on one hand, and stimulate synoviocytes to produce inflammatory factors and chemokines to enhance inflammatory responses on the other hand. This forms a vicious cycle in RA, ultimately leading to irreversible joint damage [36].

Other KEGG enriched pathways that are closely associated with RA include Th17 cell differentiation, osteoclast differentiation, Th1 and Th2 cell differentiation, and the TCR signaling pathway. Among these, Th17 cell differentiation is directly related to the immunopathological process of RA. Th17 cell differentiation leads to the production of pro-inflammatory cytokines, such as IL-1β and IL-17 [37]. These cytokines influence osteoclast differentiation and activity. Osteoclast differentiation promotes bone resorption, representing a pivotal factor in the pathogenesis of joint destruction in RA [38]. The imbalance in Th1 and Th2 cell differentiation contributes to immune dysregulation in RA. Th1 cells produce interferon-gamma (IFN-γ), promoting inflammation, while Th2 cells produce IL-4, participating in anti-inflammatory immunity. Their balance and interaction influence the overall inflammatory microenvironment in the joints of RA [39]. The TCR signaling pathway also plays a role in the activation and function of T cells. Its dysregulation may lead to exaggerated T cell activation, thereby fostering chronic inflammation and autoimmune responses, representing another significant factor in the immunopathology of RA [40].

Although our study presented up-to-date understanding of molecular mechanisms underlying anti-arthritic effects of HF and partially supported its potential as a candidate for treatment of RA, some limitations exist. First, the information from online databases is based on reviewed and predicted data; therefore, unproven and undocumented targets may not have been included in our analysis. Second, this study focused on macrophage-mediated immune inflammation in RA. However, RAFLS, T cells, osteoclasts, and other cells were also involved in the pathogenesis of RA, especially in the interactions among multiple cells which were difficult to elucidate. Establishing a three-dimensional model with multiple cell types could better simulate RA synovial tissue in vitro. Future studies on the role of HF in the IL-17 signaling pathway, in terms of anti-RA activity, will enhance our comprehension of its synergistic effects when combined with established therapeutic targets.

## Conclusion

In this study, we aimed to elucidate the underlying mechanisms of HF in treating RA using comprehensive network pharmacology and in vitro experimental validation. We preliminarily revealed that HF may suppress the activation of inflammatory macrophages by downregulating IL-17 C, p-NF-κB, and MMP9 in the IL-17 signaling pathway, providing a strong theoretical and experimental basis for follow-up research and clinical application of HF in treating the immunoinflammatory response of RA.

### Electronic supplementary material

Below is the link to the electronic supplementary material.

Supplementary Material 1

## Data Availability

The original data for this study are available by contacting the corresponding authors.

## Abbreviations

BC: Betweenness Centrality
BP: Biological Process
CCT: Closeness Centrality
CC: Cellular Component
DC: Degree Centrality
DEGs: Differentially expressed genes
DMEM: Dulbecco’s modified Eagle medium
DMSO: Dimethyl sulfoxide
EC: Eigenvector Centrality
FBS: Fetal bovine serum
GEO: Gene Expression Omnibus
GO: Gene Ontology
HF: Halofuginone hydrobromide
IC: Information Centrality
IL-17 C: Interleukin-17 C
KEGG: Kyoto Encyclopedia of Genes and Genomes
LAC: Local Average Connectivity-Based Method
LPS: Lipopolysaccharides
MF: Molecular Function
MMP-9: Matrix metalloproteinase-9
MTT: 3-[4,5-dimethyl-thiazol-2-yl]-2,5-diphenyl-tetrazolium bromide
NC: Network Centrality
OD: Optical density
OMIM: Online Mendelian Inheritance in Man
p-NF-κB: Phosphorylated nuclear factor-κB
PPI: Protein-protein interaction
RA: Rheumatoid arthritis
RAFLS: RA fibroblast-like synoviocytes
SC: Subgraph Centrality
SMILES: Simplified molecular input line entry specification
TCR: T cell receptor
