# Supplementary material for: Inhibition of IL-17 signaling in macrophages underlies the anti-arthritic effects of halofuginone hydrobromide: Network pharmacology, molecular docking, and experimental validation
Source: BMC Complement Med Ther. 2024 Feb 27;24:105. doi: 10.1186/s12906-024-04397-2 (PMC10900594; doi:10.1186/s12906-024-04397-2)
Supplement: Supplementary file 1 — Supplementary Material 1 [file 12906_2024_4397_MOESM1_ESM.docx]

Supplementary Information

Inhibition of IL-17 signaling in macrophages underlies the anti-arthritic effects of halofuginone hydrobromide: Network pharmacology, molecular docking, and experimental validation

Junping Zhu^1†^, Jiaming Wei^1†^, Ye Lin^1^, Yuanyuan Tang^1,2^, Zhaoli Su^1,3,4^, Liqing Li^3,4*^, Bin Liu^2*^ & Xiong Cai^1*^

^1^ Department of Rheumatology, First Hospital, School of Chinese Medical Sciences, Hunan University of Chinese Medicine, Changsha, 410208, Hunan, China

^2^ College of Biology, Hunan University, Changsha, 410082, Hunan, China

^3^ The Central Research Laboratory, Hunan Traditional Chinese Medical College, Zhuzhou, China

^4^ Guangxi Provincial Key Laboratory of Preventive and Therapeutic Research in Prevalent Diseases in West Guangxi, Youjiang Medical University for Nationalities, Baise, 533000, Guangxi, China

Table S1. The top 30 significant entries of GO biological process analyses

| Term ID | Term description | Gene count | Enrichment factor | Strength | Fdr |
| --- | --- | --- | --- | --- | --- |
| GO:1901700 | Response to oxygen-containing compound | 59 | 0.03765 | 0.83 | 3.60E-31 |
| GO:0010033 | Response to organic substance | 73 | 0.02424 | 0.64 | 1.29E-29 |
| GO:0042221 | Response to chemical | 83 | 0.01916 | 0.54 | 4.85E-29 |
| GO:0070887 | Cellular response to chemical stimulus | 67 | 0.02295 | 0.61 | 8.44E-25 |
| GO:1901698 | Response to nitrogen compound | 45 | 0.04206 | 0.88 | 2.49E-24 |
| GO:0050896 | Response to stimulus | 99 | 0.01230 | 0.34 | 4.17E-24 |
| GO:0010243 | Response to organonitrogen compound | 43 | 0.04357 | 0.89 | 1.02E-23 |
| GO:1901701 | Cellular response to oxygen-containing compound | 43 | 0.04076 | 0.86 | 1.17E-22 |
| GO:0001775 | Cell activation | 42 | 0.03907 | 0.85 | 2.28E-21 |
| GO:0009719 | Response to endogenous stimulus | 47 | 0.03248 | 0.77 | 2.67E-21 |
| GO:0042127 | Regulation of cell population proliferation | 49 | 0.02984 | 0.73 | 6.18E-21 |
| GO:0071310 | Cellular response to organic substance | 57 | 0.02406 | 0.64 | 6.18E-21 |
| GO:0002376 | Immune system process | 58 | 0.02338 | 0.62 | 6.71E-21 |
| GO:0045321 | Leukocyte activation | 39 | 0.04198 | 0.88 | 9.20E-21 |
| GO:0051239 | Regulation of multicellular organismal process | 64 | 0.01983 | 0.55 | 3.40E-20 |
| GO:0065008 | Regulation of biological quality | 70 | 0.01732 | 0.49 | 1.08E-19 |
| GO:0014070 | Response to organic cyclic compound | 37 | 0.04061 | 0.86 | 4.35E-19 |
| GO:0044419 | Interspecies interaction between organisms | 49 | 0.02580 | 0.67 | 1.83E-18 |
| GO:0008152 | Metabolic process | 94 | 0.01133 | 0.31 | 2.41E-18 |
| GO:0006955 | Immune response | 45 | 0.02834 | 0.71 | 4.06E-18 |
| GO:0007154 | Cell communication | 77 | 0.01447 | 0.41 | 4.06E-18 |
| GO:0048518 | Positive regulation of biological process | 82 | 0.01342 | 0.38 | 4.06E-18 |
| GO:0080134 | Regulation of response to stress | 43 | 0.02992 | 0.73 | 4.98E-18 |
| GO:0051716 | Cellular response to stimulus | 84 | 0.01294 | 0.37 | 5.28E-18 |
| GO:0048583 | Regulation of response to stimulus | 68 | 0.01653 | 0.47 | 8.09E-18 |
| GO:1901564 | Organonitrogen compound metabolic process | 76 | 0.01449 | 0.42 | 8.09E-18 |
| GO:0032101 | Regulation of response to external stimulus | 37 | 0.03653 | 0.82 | 8.80E-18 |
| GO:0071495 | Cellular response to endogenous stimulus | 39 | 0.03302 | 0.77 | 1.78E-17 |
| GO:0071704 | Organic substance metabolic process | 90 | 0.01161 | 0.32 | 2.38E-17 |
| GO:0010941 | Regulation of cell death | 45 | 0.02653 | 0.68 | 3.45E-17 |

Table S2. The top 30 significant entries of GO molecular function analyses

| Term ID | Term description | Gene count | Enrichment factor | Strength | Fdr |
| --- | --- | --- | --- | --- | --- |
| GO:0003824 | Catalytic activity | 78 | 0.01422 | 0.41 | 2.47E-17 |
| GO:0042802 | Identical protein binding | 47 | 0.02479 | 0.65 | 2.03E-16 |
| GO:0140096 | Catalytic activity, acting on a protein | 47 | 0.02221 | 0.6 | 1.04E-14 |
| GO:0036094 | Small molecule binding | 49 | 0.01948 | 0.54 | 2.17E-13 |
| GO:0005515 | Protein binding | 81 | 0.01153 | 0.32 | 2.90E-13 |
| GO:0005488 | Binding | 103 | 0.00823 | 0.17 | 3.11E-11 |
| GO:0004713 | Protein tyrosine kinase activity | 14 | 0.10219 | 1.26 | 5.29E-11 |
| GO:0097367 | Carbohydrate derivative binding | 42 | 0.01887 | 0.53 | 1.23E-10 |
| GO:0004175 | Endopeptidase activity | 20 | 0.04773 | 0.93 | 1.27E-10 |
| GO:0004672 | Protein kinase activity | 22 | 0.03873 | 0.84 | 3.65E-10 |
| GO:0043168 | Anion binding | 46 | 0.01640 | 0.47 | 5.59E-10 |
| GO:0043167 | Ion binding | 70 | 0.01131 | 0.31 | 9.08E-10 |
| GO:0004715 | Non-membrane spanning protein tyrosine kinase activity | 9 | 0.19565 | 1.55 | 4.50E-09 |
| GO:0016301 | Kinase activity | 23 | 0.03142 | 0.75 | 4.59E-09 |
| GO:0008233 | Peptidase activity | 21 | 0.03471 | 0.79 | 5.80E-09 |
| GO:0005102 | Signaling receptor binding | 32 | 0.02024 | 0.56 | 1.82E-08 |
| GO:0046914 | Transition metal ion binding | 25 | 0.02323 | 0.62 | 1.97E-07 |
| GO:0019902 | Phosphatase binding | 12 | 0.06186 | 1.05 | 2.62E-07 |
| GO:0004252 | Serine-type endopeptidase activity | 11 | 0.07143 | 1.11 | 3.01E-07 |
| GO:0000166 | Nucleotide binding | 35 | 0.01652 | 0.47 | 3.23E-07 |
| GO:0008144 | Drug binding | 9 | 0.09890 | 1.25 | 5.93E-07 |
| GO:0032559 | Adenyl ribonucleotide binding | 28 | 0.01840 | 0.52 | 1.84E-06 |
| GO:0016787 | Hydrolase activity | 36 | 0.01488 | 0.43 | 2.10E-06 |
| GO:0019903 | Protein phosphatase binding | 10 | 0.06711 | 1.08 | 2.10E-06 |
| GO:0019899 | Enzyme binding | 34 | 0.01519 | 0.44 | 3.45E-06 |
| GO:0031406 | Carboxylic acid binding | 11 | 0.05046 | 0.96 | 5.96E-06 |
| GO:0019842 | Vitamin binding | 9 | 0.06338 | 1.06 | 1.54E-05 |
| GO:0019900 | Kinase binding | 18 | 0.02426 | 0.64 | 1.71E-05 |
| GO:0032555 | Purine ribonucleotide binding | 29 | 0.01556 | 0.45 | 2.58E-05 |
| GO:0005524 | ATP binding | 25 | 0.01708 | 0.49 | 3.62E-05 |

Table S3. The top 30 significant entries of GO cellular component analyses

| Term ID | Term description | Gene count | Enrichment factor | Strength | Fdr |
| --- | --- | --- | --- | --- | --- |
| GO:0005576 | Extracellular region | 59 | 0.01416 | 0.41 | 5.43E-11 |
| GO:0005615 | Extracellular space | 52 | 0.01628 | 0.47 | 5.43E-11 |
| GO:0031982 | Vesicle | 57 | 0.01469 | 0.42 | 5.43E-11 |
| GO:1904813 | ficolin-1-rich granule lumen | 14 | 0.11200 | 1.3 | 5.43E-11 |
| GO:0030141 | Secretory granule | 26 | 0.03077 | 0.74 | 3.38E-10 |
| GO:0005737 | Cytoplasm | 97 | 0.00849 | 0.18 | 6.14E-10 |
| GO:0031410 | Cytoplasmic vesicle | 42 | 0.01760 | 0.5 | 6.38E-10 |
| GO:0070062 | Extracellular exosome | 39 | 0.01858 | 0.52 | 8.56E-10 |
| GO:0031983 | Vesicle lumen | 17 | 0.05152 | 0.97 | 1.07E-09 |
| GO:0099503 | Secretory vesicle | 27 | 0.02673 | 0.68 | 1.18E-09 |
| GO:0034774 | Secretory granule lumen | 16 | 0.04938 | 0.95 | 7.03E-09 |
| GO:0009986 | Cell surface | 23 | 0.02791 | 0.7 | 1.94E-08 |
| GO:0045121 | Membrane raft | 14 | 0.04321 | 0.89 | 5.19E-07 |
| GO:0005829 | Cytosol | 56 | 0.01078 | 0.29 | 2.77E-06 |
| GO:0031012 | Extracellular matrix | 16 | 0.03036 | 0.74 | 3.92E-06 |
| GO:0005886 | Plasma membrane | 56 | 0.01054 | 0.28 | 5.92E-06 |
| GO:0036021 | Endolysosome lumen | 4 | 0.80000 | 2.16 | 7.78E-06 |
| GO:0043235 | Receptor complex | 13 | 0.03412 | 0.79 | 1.84E-05 |
| GO:0062023 | Collagen-containing extracellular matrix | 13 | 0.03283 | 0.77 | 2.71E-05 |
| GO:0098590 | Plasma membrane region | 22 | 0.01805 | 0.51 | 6.12E-05 |
| GO:0030054 | Cell junction | 29 | 0.01398 | 0.4 | 0.00015 |
| GO:0031904 | Endosome lumen | 5 | 0.14286 | 1.41 | 0.00015 |
| GO:0070161 | Anchoring junction | 17 | 0.02073 | 0.57 | 0.00019 |
| GO:0012505 | Endomembrane system | 47 | 0.01035 | 0.27 | 0.0002 |
| GO:0045177 | Apical part of cell | 12 | 0.02857 | 0.71 | 0.00025 |
| GO:0005775 | Vacuolar lumen | 8 | 0.04598 | 0.92 | 0.00039 |
| GO:0043227 | Membrane-bounded organelle | 90 | 0.00724 | 0.11 | 0.00053 |
| GO:0030424 | Axon | 14 | 0.02167 | 0.59 | 0.00078 |
| GO:1904724 | Tertiary granule lumen | 5 | 0.09091 | 1.21 | 0.00091 |
| GO:0070013 | Intracellular organelle lumen | 53 | 0.00905 | 0.21 | 0.0015 |

Table S4. The top 30 significant entries of KEGG pathway enrichment analyses

| Term ID | Pathway | Gene count | Enrichment factor | Strength | Fdr |
| --- | --- | --- | --- | --- | --- |
| hsa05200 | Pathways in cancer | 20 | 0.03868 | 0.84 | 4.7E-09 |
| hsa04657 | IL-17 signaling pathway | 10 | 0.10870 | 1.29 | 2.8E-08 |
| hsa05135 | Yersinia infection | 11 | 0.08800 | 1.20 | 2.8E-08 |
| hsa05215 | Prostate cancer | 10 | 0.10417 | 1.27 | 2.8E-08 |
| hsa05235 | PD-L1 expression and PD-1 checkpoint pathway in cancer | 10 | 0.11364 | 1.31 | 2.8E-08 |
| hsa04659 | Th17 cell differentiation | 10 | 0.09901 | 1.25 | 3.24E-08 |
| hsa04062 | Chemokine signaling pathway | 12 | 0.06452 | 1.06 | 4.95E-08 |
| hsa05205 | Proteoglycans in cancer | 12 | 0.06122 | 1.04 | 7.6E-08 |
| hsa04380 | Osteoclast differentiation | 10 | 0.08197 | 1.17 | 1.14E-07 |
| hsa04658 | Th1 and Th2 cell differentiation | 9 | 0.10345 | 1.27 | 1.14E-07 |
| hsa04621 | NOD-like receptor signaling pathway | 11 | 0.06322 | 1.05 | 1.99E-07 |
| hsa04660 | T cell receptor signaling pathway | 9 | 0.08911 | 1.20 | 2.87E-07 |
| hsa04917 | Prolactin signaling pathway | 8 | 0.11594 | 1.32 | 2.87E-07 |
| hsa05230 | Central carbon metabolism in cancer | 8 | 0.11594 | 1.32 | 2.87E-07 |
| hsa05161 | Hepatitis B | 10 | 0.06289 | 1.05 | 7.69E-07 |
| hsa05152 | Tuberculosis | 10 | 0.05952 | 1.03 | 1.18E-06 |
| hsa04926 | Relaxin signaling pathway | 9 | 0.07031 | 1.10 | 1.38E-06 |
| hsa01100 | Metabolic pathways | 26 | 0.01797 | 0.51 | 1.69E-06 |
| hsa01522 | Endocrine resistance | 8 | 0.08421 | 1.18 | 1.86E-06 |
| hsa04151 | PI3K-Akt signaling pathway | 13 | 0.03714 | 0.82 | 1.86E-06 |
| hsa04620 | Toll-like receptor signaling pathway | 8 | 0.07921 | 1.15 | 2.62E-06 |
| hsa05120 | Epithelial cell signaling in Helicobacter pylori infection | 7 | 0.10448 | 1.27 | 2.67E-06 |
| hsa04931 | Insulin resistance | 8 | 0.07477 | 1.13 | 3.62E-06 |
| hsa04935 | Growth hormone synthesis, secretion and action | 8 | 0.06780 | 1.09 | 0.000007 |
| hsa05131 | Shigellosis | 10 | 0.04587 | 0.92 | 7.55E-06 |
| hsa05323 | Rheumatoid arthritis | 7 | 0.08235 | 1.17 | 1.01E-05 |
| hsa05418 | Fluid shear stress and atherosclerosis | 8 | 0.06154 | 1.04 | 1.24E-05 |
| hsa04210 | Apoptosis | 8 | 0.06061 | 1.04 | 1.34E-05 |
| hsa04915 | Estrogen signaling pathway | 8 | 0.06015 | 1.03 | 1.36E-05 |
| hsa05167 | Kaposi sarcoma-associated herpesvirus infection | 9 | 0.04813 | 0.94 | 1.63E-05 |

Table S5. Detailed information on the topological networks of the top 10 targets

| NO. | Target | BC | CC | DC | SC | EC | IC | LAC | NC |
| --- | --- | --- | --- | --- | --- | --- | --- | --- | --- |
| 1 | EGFR | 1978.18370 | 0.62048 | 48 | 9392489 | 0.29888 | 6.36019 | 10.87500 | 34.83101 |
| 2 | MMP9 | 1010.04736 | 0.57865 | 41 | 7132474 | 0.26041 | 6.22805 | 10.78049 | 30.61889 |
| 3 | TLR4 | 1369.08640 | 0.56906 | 36 | 5245888 | 0.22336 | 6.10801 | 8.44445 | 21.42984 |
| 4 | ESR1 | 526.96480 | 0.52821 | 31 | 4647423 | 0.21024 | 5.95730 | 8.96774 | 18.05450 |
| 5 | MMP2 | 299.64627 | 0.52821 | 27 | 4113814 | 0.19776 | 5.80587 | 10.00000 | 17.38487 |
| 6 | PPARG | 1522.30920 | 0.57222 | 35 | 3972675 | 0.19438 | 6.08066 | 6.91429 | 20.46655 |
| 7 | MAPK1 | 317.44623 | 0.53093 | 25 | 3669718 | 0.18680 | 5.71644 | 8.96000 | 13.55388 |
| 8 | JAK2 | 415.12137 | 0.52551 | 24 | 3411094 | 0.18010 | 5.66755 | 8.33333 | 12.34797 |
| 9 | STAT1 | 121.12589 | 0.51244 | 21 | 3222984 | 0.17506 | 5.50077 | 9.71429 | 12.61023 |
| 10 | MAPK8 | 130.68907 | 0.50739 | 20 | 3184220 | 0.17402 | 5.43728 | 9.10000 | 11.05643 |

Table S6. Detailed information of core targets and their binding energy with HF

| NO. | Core targets | Information | Binding energy (kcal/mol) |
| --- | --- | --- | --- |
| 1 | MMP9 | Matrix metalloproteinase-9; May play an essential role in local proteolysis of the extracellular matrix and in leukocyte migration. Could play a role in bone osteoclastic resorption. Cleaves KiSS1 at a Gly-\|-Leu bond. Cleaves type IV and type V collagen into large C-terminal three quarter fragments and shorter N-terminal one quarter fragments. Degrades fibronectin but not laminin or Pz-peptide. | -9.1 |
| 2 | EGFR | Epidermal growth factor receptor; Receptor tyrosine kinase binding ligands of the EGF family and activating several signaling cascades to convert extracellular cues into appropriate cellular responses. Known ligands include EGF, TGFA/TGF-alpha, amphiregulin, epigen/EPGN, BTC/betacellulin, epiregulin/EREG and HBEGF/heparin-binding EGF. | -8.1 |
| 3 | ESR1 | Estrogen receptor; Nuclear hormone receptor. The steroid hormones and their receptors are involved in the regulation of eukaryotic gene expression and affect cellular proliferation and differentiation in target tissues. Ligand-dependent nuclear transactivation involves either direct homodimer binding to a palindromic estrogen response element (ERE) sequence or association with other DNA- binding transcription factors, such as AP-1/c-Jun, c-Fos, ATF-2, Sp1 and Sp3, to mediate ERE-independent signaling. | -8.0 |
| 4 | MAPK1 | Mitogen-activated protein kinase 1; Serine/threonine kinase which acts as an essential component of the MAP kinase signal transduction pathway. MAPK1/ERK2 and MAPK3/ERK1 are the 2 MAPKs which play an important role in the MAPK/ERK cascade. They participate also in a signaling cascade initiated by activated KIT and KITLG/SCF. Depending on the cellular context, the MAPK/ERK cascade mediates diverse biological functions such as cell growth, adhesion, survival and differentiation through the regulation of transcription, translation, cytoskeletal rearrangements. | -7.8 |
| 5 | JAK2 | Tyrosine-protein kinase JAK2; Non-receptor tyrosine kinase involved in various processes such as cell growth, development, differentiation or histone modifications. Mediates essential signaling events in both innate and adaptive immunity. In the cytoplasm, plays a pivotal role in signal transduction via its association with type I receptors such as growth hormone (GHR), prolactin (PRLR), leptin (LEPR), erythropoietin (EPOR), thrombopoietin (THPO); or type II receptors including IFN-alpha, IFN-beta, IFN-gamma and multiple interleukins. | -7.8 |
| 6 | MMP2 | Matrix metalloproteinase-2 (gelatinase a); 72 kDa type IV collagenase; Ubiquitinous metalloproteinase that is involved in diverse functions such as remodeling of the vasculature, angiogenesis, tissue repair, tumor invasion, inflammation, and atherosclerotic plaque rupture. As well as degrading extracellular matrix proteins, can also act on several nonmatrix proteins such as big endothelial 1 and beta-type CGRP promoting vasoconstriction. Also cleaves KISS at a Gly-\|-Leu bond. Appears to have a role in myocardial cell death pathways. | -7.6 |
| 7 | PPARG | Peroxisome proliferator-activated receptor gamma; Nuclear receptor that binds peroxisome proliferators such as hypolipidemic drugs and fatty acids. Once activated by a ligand, the nuclear receptor binds to DNA specific PPAR response elements (PPRE) and modulates the transcription of its target genes, such as acyl-CoA oxidase. It therefore controls the peroxisomal beta-oxidation pathway of fatty acids. Key regulator of adipocyte differentiation and glucose homeostasis. ARF6 acts as a key regulator of the tissue-specific adipocyte P2 (aP2) enhancer. | -7.6 |
| 8 | MAPK8 | Mitogen-activated protein kinase 8/9/10 (c-jun n-terminal kinase); Mitogen-activated protein kinase 8; Serine/threonine-protein kinase involved in various processes such as cell proliferation, differentiation, migration, transformation and programmed cell death. Extracellular stimuli such as proinflammatory cytokines or physical stress stimulate the stress-activated protein kinase/c-Jun N-terminal kinase (SAP/JNK) signaling pathway. In this cascade, two dual specificity kinases MAP2K4/MKK4 and MAP2K7/MKK7 phosphorylate and activate MAPK8/JNK1. | -7.2 |
| 9 | TLR4 | Toll-like receptor 4; Cooperates with LY96 and CD14 to mediate the innate immune response to bacterial lipopolysaccharide (LPS). Acts via MYD88, TIRAP and TRAF6, leading to NF-kappa-B activation, cytokine secretion and the inflammatory response. Also involved in LPS-independent inflammatory responses triggered by free fatty acids, such as palmitate, and Ni(2+). Responses triggered by Ni(2+) require non-conserved histidines and are, therefore, species-specific. | -7.1 |
| 10 | STAT1 | Signal transducer and activator of transcription 1-alpha/beta; Signal transducer and transcription activator that mediates cellular responses to interferons (IFNs), cytokine KITLG/SCF and other cytokines and other growth factors. Following type I IFN (IFN-alpha and IFN-beta) binding to cell surface receptors, signaling via protein kinases leads to activation of Jak kinases (TYK2 and JAK1) and to tyrosine phosphorylation of STAT1 and STAT2. The phosphorylated STATs dimerize and associate with ISGF3G/IRF-9 to form a complex termed ISGF3 transcription factor, that enters the nucleus. | -6.2 |
